# Prevalence of Self-reported Domestic Elder Abuse and Its Relation with Personality Traits of Older People and Their Family Caregivers

**DOI:** 10.34172/aim.28107

**Published:** 2024-05-25

**Authors:** Shahin Salarvand, Zahra Azizi, Saeid Bitaraf, Nahid Momeni-Safarabad

**Affiliations:** ^1^Hepatitis Research Center, Faculty of Nursing and Midwifery, Lorestan University of Medical Sciences, Khorramabad, Iran; ^2^Student Research Committee, Faculty of Nursing and Midwifery, Lorestan University of Medical Sciences, Khorramabad, Iran; ^3^Epidemiology, Clinical Sciences Research Institute, Ahvaz Jundishapur University of Medical Sciences, Ahvaz, Iran; ^4^Faculty of Medicine, Lorestan University of Medical Sciences, Khorramabad, Iran

**Keywords:** Elder abuse, Elder mistreatment, Family caregiver, Personality traits, Older people

## Abstract

**Background:**

Elder abuse (EA) is a serious public health issue recognized as a healthcare priority. Personality traits can influence social behaviors. This study aimed to determine the prevalence of self-reported domestic EA and its relationship with personality traits of older people and their family caregivers.

**Methods:**

A cross-sectional study was conducted in 2022. The research population included older people living in the urban community of the Lorestan Province (in the western region of Iran) selected by multistage cluster sampling. In general, 998 older people and their family caregivers were sampled. The data collection tool was a three-part questionnaire: a. demographic characteristics of the older people, b. questionnaire on the incidence of elder abuse, and c. short version of the NEO Five-Factor Inventory-Revised (NEO-FFI-R) for measuring the personality traits of the older people or family caregivers. The statistical software used was Stata 14.

**Results:**

The present study reported that the prevalence of EA at home was 37.78%. In the present study, older age, female gender, unmarried/single status, lower education, unemployment, and rented house characteristics were predictors of EA. High agreeableness, high extroversion, and low neuroticism reduce conflict and tension in older people with their relatives and family, which appear to be protective factors against EA.

**Conclusion:**

Policymakers and health experts should prepare training and screening programs to consider these factors so that older people exposed to EA can be identified more quickly and early interventions can be used to improve their health status and increase their quality of life.

## Introduction

 The increasing day-to-day prevalence of the older population worldwide highlights the need to pay attention to their issues.^1^ Eighty percent of the one million people who turn 60 every month live in developing countries.^2^ This growing population creates many challenges, including abuse and neglect of older people,^3^ and paying attention to their quality of life is a high priority. The suffering of older people from annoying and neglectful behavior is one of the most critical obstacles to improving quality of life in healthy aging individuals.^4^

 Elder abuse (EA) is a severe public health issue that has been recognized as a healthcare priority.^5,6^ It can have serious consequences, such as premature mortality,^7-9^ poverty, cognitive decline, depression, physical injuries, hospitalization, and institutionalized long-term care facilities.^9-11^ Therefore, EA is not only a social issue but also a medical problem.^12^ However, this phenomenon is under-recognized and has received little attention^12,13^ because it is highly complex and multifactorial.^14^

 There is no consensus definition of EA in the literature, which leads to different risk estimations.^15,16^ Abuse refers to the infliction of pain and suffering on older people, which may occur through an insult or intentional or unintentional failure to take necessary measures.^11,17^ The definition of “EA” by the World Health Organization (WHO) is “a single or repeated act or lack of appropriate action occurring within any relationship in which there is an expectation of trust that causes harm or distress to an older person”.^18^ Five types of EA—psychological/emotional, physical, sexual, financial, and unintentional/intentional forms of neglect—have been recognized.^19,20^

 Despite the increasing reports of EA every year^21^ and its high prevalence according to many studies from around the world,^22,23^ the measurement of its prevalence and research are difficult because EA is a complex and underreported phenomenon because EA is often naturally invisible/hidden mistreatment.^5,24-27^ This means that EA is unrecognizable even by the victims themselves,^26^ and persons whom older people rely on them often perpetrate abuse against vulnerable older people.^24^ EA exists in families, but for cultural reasons, it remains hidden from public opinion.^28^ In other words, EA is assumed to be a private matter within the family, often leading to underreporting of EA in the community.^20^ Therefore, despite its prevalence and severity, EA remains a neglected global public health priority, receiving little attention from national and international governments and organizations^29-32^ and having few resources.^30^

 A better understanding of the vastness and complexity of this problem is essential for preventing it.^33^ The frequency of elder neglect and abuse varies widely. Studies have reported that the incidence of EA and neglect ranges from 2.2 to 62.3% internationally.^15,34-39^ In Iran, similar to many countries, various studies have been conducted in this area that reported the prevalence of EA to range from 17.1% to 90.4% in different regions.^4,28,40-46^

 Most EA occurs at home by family members/caregivers.^12,47-48^ Identifying older people and caregiver risk factors associated with EA is highly important for the prevention and management of such abuse.^23^ Personality traits play an important role in defining people’s cognition and behavior,^49^ and personality traits are significantly related to psychological outcomes.^50^ Individual differences, such as personality traits, may modify the response to care.^51^ It has been proven that different personality traits can influence emotions, decision-making, and social behaviors.^52^ Personality is a specific way of thinking, feeling, and behaving; it includes moods, attitudes, and beliefs, and is clearly expressed in interactions with other people. It has behavioral, inherent, and acquired characteristics that distinguish one person from another and can be seen in the relationships of people with the environment and social groups.^53^ The five main personality traits include neuroticism (N), extraversion (E), openness (O), agreeableness (A), and conscientiousness (C).^54^

 Although studies have been conducted on the relationship between older people’s personality traits and the incidence of EA,^55^ which have not reported the same findings, caregiver risk factors may be more amenable to change and should receive more attention from healthcare professionals. A review of the EA literature reveals lack of knowledge about the relationship between caregivers’/older people’s personality traits and taking-place EA.^44,56-58^ Two studies have addressed only neurotic personality traits for caregivers^59^ and only five or two dimensions of elderly people’s personality traits and EA.^55,60^ In addition, a literature review indicated that culture or ethnicity determines how EA is defined, manifested, perceived, and reported.^61-67^ There are similar and culturally unique findings among Asian countries in studies conducted on EA by caregivers.^23,68^ In most Asian societies, there is a solid traditional notion of filial piety or filial obligation, in which children must look after their parents. This notion is a significant reason that Asian countries have generally been slower than Western countries in addressing and responding to the issue of elder abuse; there is a strong supposition that senior parents are well taken care of by adult children (filial obligation embedded in Confucianism^61^ and Quranic teachings^69^) which could prevent issues of EA. As mentioned above, the relationship between the perpetrators of EA and the victims of abuse is very complex. EA can occur in any situation or by anyone in a position of trust.^70^ Unfortunately, the literature provides little information about perpetrators and their motivations for EA.^71^

 Moreover, no such study has been conducted in our province or country, and there is little knowledge about the relationship between caregiver or elder personality traits and the incidence of EA. This study aimed to determine the prevalence of self-reported domestic EA and its relationship with personality traits of older people and their family caregivers.

## Materials and Methods

 A cross-sectional study was conducted over a 6-month period from May to November 2022. The research population included older people living in the urban community of the Lorestan Province (a province in the western region of Iran) who were selected by multistage cluster sampling. First, three cities were chosen randomly from among the cities of the Lorestan province; then, four healthcare centers were randomly selected from the list of health centers in each chosen city. Afterwards, 41 or 42 older adults were selected from among the recorded older adults in each health center by a simple random sampling method. The sample size was determined according to the systematic review and meta-analysis article conducted by Yon et al^34^ to investigate the prevalence of elder abuse. The prevalence of this problem was calculated to be 15.7%, considering an accuracy equivalent to 25% of the overall prevalence and using the sample size formula. Considering an effect size or design effect of 1.25, the loss of 20% of the elderly, the sample size was found to be approximately 499 older people/family caregivers. In general, 998 older people and their family caregivers were sampled.


$$n=\frac{z_{1-\alpha /2}^{2}\times p\left(1-p\right)}{d^{2}}$$

###  Eligibility Criteria

 We included patients aged 60 years and older. The participants were screened for lack of cognitive impairment according to the six cognitive impairment test scores (6-CIT)^72^ and for functional independence of older people according to self-reports of older people and observation by the researcher. Before entering the study, older people were screened for cognitive and functional status. This means that older adults with cognitive and physical disabilities were omitted because research studies have shown that they are abused by their caregivers more than their healthy counterparts.^73^ For this purpose, after selecting older people, the 6-CIT was used to screen the cognitive status of older people, and older people who were in the normal range in terms of cognitive impairment and observed/self-reported functional status were included in the study to determine the effect of the personality traits of the caregiver and older people on the occurrence of EA. The exclusion criterion for older people and family caregivers was incomplete completion of the questionnaire.

 The second author called from the health care center to independently invite older people. After the older adult came to the center by himself, measures such as measuring blood pressure and blood sugar were performed. She tried to establish trust-based communication with elders. Then, to measure the cognitive status of older people, 6-CIT was used. If there was no cognitive impairment, the older people entered the study after providing informed and written consent. All questionnaires were completed via face-to-face interviews by the second author.

 In the next step, to complete the questionnaire on the personality traits of the caregiver, the second author went to the doors of the older adults’ homes and filled out the caregiver personality traits questionnaire. The primary family caregiver was the head of the household or older people.

###  Data Collection

 The data collection tool used was a three-part questionnaire: a. demographic characteristics of older people, b. questionnaire on the incidence of elder abuse, and c. short version of the NEO Five-Factor Inventory-Revised (NEO-FFI-R) for measuring the personality traits of older people/family caregivers. Considering the illiteracy or low literacy of most older people, education was divided into two categories: literate and illiterate.

 The Domestic Elder Abuse Questionnaire was developed in Persian by Heravi and colleagues in 2010.^74^ This questionnaire examines family misbehavior toward older people. This questionnaire has 49 questions, and its purpose is to examine family misbehavior toward older people on eight subscales: neglect of care (items 33-43), psychological abuse (items 1-8), physical abuse (items 19-22), financial abuse (items 23-28), deprivation of authority (items 9-18), rejection (items 29-32), financial neglect (items 46-49), and emotional abuse (items 44-45).^75^ The questionnaire is scored on a 3-point Likert scale. The items mentioned above have the options “no case (0)”, “no (1)”, and “yes (2)”. The choice of “no case” means that the desired phrase does not agree with the living conditions of older people. To obtain the score for each dimension, the total scores for all dimension questions are summed together.^74^ The obtained scores are in the range of 0 to 100; there is no cutoff point, and a higher score indicates greater severity of elder abuse.^75^ To obtain the scores for each mentioned subscale, the total items of that subscale are summed. The calculated Cronbach’s alpha coefficient (above 0.97 for all dimensions) and test-retest stability (0.99) confirmed the excellent reliability of the questionnaire. Health service providers, including nurses, are suitable agents for investigating the mistreatment of older people in Iranian families because of the appropriate reliability, validity, and applicability of these tools in different situations.^74^

 The short version of the NEO-FFI-R comprises 60 items (12 per trait) that are appraised using a Likert scale ranging from 1 (completely disagree) to 5 (completely agree). Given that a total score is not received from this instrument, five scores are accepted, and each is related to a personality trait. Higher scores indicate greater intensity of a particular personality trait.^76^ It provides a concise and comprehensive measure of five personality traits (neuroticism, extroversion, openness to experience, agreeableness, and conscientiousness) in different contexts.^77^ The participants’ neuroticism, which includes stress, mood swings, and anxiety, was obtained from the following items: 1(R)^[1]^, 6, 11, 16, 21, 26, 31, 36, 41, 46(R), 51, and 56.^78^ A score of 12-24 indicates that high emotional stability is associated with a high probability of a lack of emotional problems such as depression and anxiety. Scores of 25-48 suggest that the person is in a moderate state. Scores of 49-60 suggest unpleasant emotions such as sadness, anxiety, and anger.

 Extraversion involves talkativeness, high energy, and high productivity. Personality traits such as broad interest, insight, and strong imagination are sub-items of openness and are obtained from the following items: 2, 7, 12(R), 17, 22, 27(R), 32, 37, 42(R), 47, 52, 57(R).^78^ A score of 12-24 indicates that the person has an introversion trait; she/he enjoys solitude more often and stays away from crowds and other people. A score of 25-48 indicates that the person is in a moderate state in terms of introversion and extroversion. Scores of 49-60 indicate that the person is an extrovert, shares her/his emotions with others more easily, and prefers to be with others.

 Information on agreeableness, empathy, and kindness was obtained from the following items: 4, 9(R), 14(R), 19, 24(R), 29(R), 34, 39(R), 44(R), 49, 54(R), and 59.^78^ Scores of 12-24 indicate that the person is less compatible with those around them. This issue can cause them to experience more interpersonal conflict. Scores of 25-48 indicate a moderate degree of agreeableness. Scores of 49-60 suggest that the person can adapt well to others and is highly agreeable.

 Conscientiousness, which is composed of perfectionism, organization, and decision-making ability,^79^ is obtained from the following items: 5, 10, 15(R), 20, 25, 30(R), 35,40, 45(R), 50, 55(R), and 60.^78^ Scores of 12-24 indicate that the person often has a problem following the law. This issue can also affect their job and social performance and eventually become a problem for them. Scores of 25-48 indicate that the person is not so irresponsible or law-abiding, but he still has a problem with this issue to some extent. Therefore, he may sometimes feel that he cannot plan well for himself and obey the existing rules. Scores of 49-60 indicate that the respondent is a responsible, law-abiding, and orderly person. It is also likely that he/she has a high ability to plan.

 Openness to experience was obtained from the following items: 3(R), 8(R), 13, 18(R), 23(R), 28, 33(R), 38(R), 43, 48(R), 53, and 58.^78^ Scores of 12-24 indicate that the person is most likely a conservative person. Therefore, he does not take many risks and does not welcome new experiences. Scores of 25-48 indicate that he/she is not conservative, but he/she is not very interested in new topics either. In fact, it can be said that their openness to new experiences is balanced. Scores of 49-60 indicate that he/she usually welcomes anything new.

 This questionnaire has been psychometrically assessed by different researchers in Iran.^80-84^ This instrument was completed by older people and their family caregivers. The Cronbach’s alpha coefficient in its subscales for older people was above 0.73, and for family caregivers, it was above 0.70.

###  Data Analysis

 The total weight is calculated from the multiplication of three weights: 1. The weights related to cluster 2. The weight of each participant in cluster 3. The weights for gender and age groups differing between the population and the sample. The total weight was calculated by inverse multiplication of the mentioned weights.

 In the present study, the prevalence of elder abuse and its relationship with the personality traits of older people were determined using survey data analysis. To investigate the relationships between elder abuse and personality traits, odds ratios were calculated via logistic regression. The statistical software used was Stata 14.

## Results

 In the present study, two groups of older people and their family caregivers participated. According to the descriptive statistics of the older people samples, elderly females constituted 43.3% and elderly males constituted 56.7% of the older people sample. Additionally, out of 499 older people, 37.78% (31.23–44.32) reported abuse. A total of 499 older people (216 females and 283 males) with a mean age of 70.10 ± 8.18 years participated in this study. Most of the participants were married (402, 80.56%), and 432 (86.57%) of them had their own house. Most of them were illiterate (317, 63.52%).

 The results showed that there were significant relationships between age, gender, employment status, education, marital status, type of residence and the experience of EA among the family caregivers (Table 1). This means that older old people, female in age, have experienced more abuse by caregivers in their families. Compared with male older people, female older people were more exposed to family caregiver EA. Additionally, unemployed older people, housewives, self-employed individuals, and retired people were exposed to caregiver abuse in the family in descending order. Notably, unemployed older people were most likely to be exposed to abuse, and retired older people had experienced caregiver abuse less than others had. The illiterate older people experienced caregiver abuse in the family more than their educated counterparts did.

**Table 1:** Relationships between the Demographic Characteristics of Older People and Self-Reported Experiences of Domestic Abuse

**Demographic Characteristics**		**Number (%)**	**With the Experienced Abuse**	**Without the Experienced Abuse**	**The Spearman Correlation Coefficient**	***P*** Value*
			**N=223**	**N=276**		
Gender	Female	216 (43.3%)	122 (54.7%)	94 (34.1%)	0.57	< 0.001
	Male	283 (56.7%)	101 (45.3%)	182 (65.9%)		
Age	60-64	140 (28.06%)	29 (13.00%)	111 (40.22%)	0.38	< 0.001
	65-69	135 (27.05%)	50 (22.42%)	85 (30.80%)		
	70-74	76 (15.23%)	39 (17.49%)	37 (13.41%)		
	75-79	66 (13.23%)	43 (19.28%)	23 (8.33%)		
	80 ≤	82 (16.43%)	62 (27.80%)	20 (7.25%)		
Employment status	Self- employment	182 (36.47%)	56 (25.1%)	126 (45.7%)	0.62	< 0.001
	Retirement	63 (12.62%)	9 (4.0%)	54 (19.6%)		
	Unemployed	117 (23.44%)	80 (35.9%)	37 (13.4%)		
	Housekeeper	137 (27.45%)	78 (35.0%)	59 (21.4%)		
Education	Illiterate	317 (63.52%)	180 (80.7%)	137 (49.6%)	0.32	< 0.001
	Literate	182 (36.47%)	43 (19.3%)	139 (50.4%)		
Marital status	Married	402 (80.56%)	151 (67.7%)	251 (91.3%)	0.49	< 0.001
	Single	96 (19.23%)	72 (32.3%)	24 (8.7%)		
Type of residence	Owner	432 (86.57%)	178 (79.8%)	254 (92.7%)	0.35	< 0.001
	Tenant	65 (13.03%)	45 (20.2%)	20 (7.3%)		

*P value ˂ 0.05 is significant

 Unmarried older people reported more experiences of abuse by caregivers. Older people who were renting tenants experienced much more abuse from their caregivers than did their counterparts who owned houses.

 In all types of EA, in general, the frequency of older people who had not experienced any type of abuse was greater than that of older people who had experienced abuse; in other words, 37.78% (31.23-44.32) of all older people participating in the current study had experienced abuse in some way. According to the findings in Table 2, psychological abuse had the highest frequency among all types of EA experienced by older people (25.13%). After that, other types, in descending order of frequency, such as financial abuse (21.76%), deprivation of authority (20.37%), neglect of care (14.89%), emotional abuse (13.82%), financial neglect (10.25%), physical abuse (7.05%), and rejection (6.81%), were reported by older people in this study (Table 2).

**Table 2:** Total Percentage and Type of Elder Abuse

**Abuse type **	**Percent**	**95% CI**
Psychological abuse	25.13	19.38-30.88
Physical abuse	7.05	10.39-3.72
Financial abuse	21.76	27.14-16.38
Rejection	6.81	10.02-3.60
Care neglect	14.89	19.51-10.27
Emotional abuse	13.82	18.27-9.36
Financial neglect	10.25	8.29-12.21
Deprivation of choice	20.37	15.09-25.64
Total percent of elder abuse	37.78	31.23-44.32

CI, confidence interval.

 In the present study, assessment of the personality traits of older people and their family caregivers showed that most of the participants in both groups had an average level of neuroticism, which means that they have an average level of emotional stability; as a result, they may sometimes experience unpleasant emotional conditions; of course, these conditions are often transient. A small number of participants were in the normal range, which means that only a limited number of older people/caregivers have very high emotional stability; as a result, they are most likely not to experience emotional disorders such as depressive episodes (Table 3).

**Table 3:** Frequency of Older People and Family Caregiver Personality Trait Types and Univariate Correlation of Total Elder Abuse with these Traits

**Personality traits**		**Elder**				**Family caregiver**			
		**No. (%) of Personality Traits**	**Elder Abuse**			**No. (%) of Personality Traits**	**Elder Abuse**		
			**OR**	***P*** ** Value**	**CI (Min–Max)**		**OR**	***P*** ** Value**	**CI (Min–Max)**
Neuroticism	Normal/High emotional stability (12-24)	4 (0.80%)	12.07	0.03	1.37-106.42	3(0.06%)	8.63	0.13	0.54-137.79
	Average state of emotional stability (25-48)	495 (99.20%)				496(99.4%)			
	Unpleasant emotions (49-60)	0 (0.0%)				0(0.0%)			
Extraversion	Introverted (12-24)	1 (0.20%)	6.07	0.01	1.60-23.03	0(0.0%)	0.14	0.04	0.02- 0.90
	Medium (25-48)	484 (96.99%)				492(98.6%)			
	Extroverted (49-60)	14 (2.81%)				7(1.4%)			
Openness to experience	Conservative (12-24)	0 (0.0%)	1.00			0(0.0%)	1.00		
	Medium (25-48)	499 (100%)				499(100%)			
	Extreme welcome (49-60)	0 (0.0%)				0(0.0%)			
Agreeableness	Incompatibility (12-24)	16 (3.21%)	0.20	0.01	0.06-0.65	17(3.41%)	0.44	0.34	0.08-2.36
	Medium (25-48)	483 (96.79%)				482(96.59%)			
	Too much compatibility (49-60)	0 (0.0%)				0(0.0%)			
Conscientiousness	Evading the law (12-24)	0 (0.0%)	0.55	0.22	0.22-1.42	40(8.02%)	0.66	0.44	0.23-1.89
	Medium (25-48)	455 (91.18%)				452(91.98%)			
	Subject to the rules (49-60)	44 (8.82%)				0(0.0%)			

 The present study showed that there is an inverse and significant relationship between the personality traits of neuroticism and extroversion, conscientiousness, and agreeableness. This means that the greater the number of traits is, the less neuroticism there is, the greater the emotional stability is, and the lower the probability of EA.

 In the extraversion subscale, the findings indicated that most of the older people/caregivers who participated in this study were in a moderate state in terms of extraversion or introversion. This means that older people/caregivers with this type of personality prefer loneliness in some situations and do not like to spend time alone in other situations. A small group of older people/caregivers also had an extroverted personality; in other words, they preferred to be with a group, and ultimately, a limited number of them had an introverted personality and stayed away from being in a crowded area (Table 3).

 Additionally, in the present study, there was an inverse and significant relationship between extraversion and neuroticism in caregivers/older people, which indicated that the greater the extraversion score was in older people/caregivers, the lower the neuroticism was in older people/caregivers and vice versa (Table 4). Because extroverted people share their emotions with others more easily and prefer to be with a group compared with people with loneliness, it is certain that relationships become more transparent and that demands are easily expressed; therefore, it can be said that the occurrence of EA decreases.

**Table 4:** Multivariable Correlation of the Relationship between Total Elder Abuse and the Personality Traits of Older People and their Family Caregivers (After Controlling for the Variables Age, Sex, Employment Status, Education Level, and Type of Residence)

**Personality Traits**		**Elder Abuse**		
		**OR**	***P*** ** Value**	**CI (Min–Max)**
Elder	Neuroticism	4.23	0.16	0.56-32.08
	Extraversion	1.88	0.38	0.45-7.74
	Openness to experience			
	Agreeableness	0.09	0.18	0.00-2.98
	Conscientiousness	0.88	0.84	0.26-3.04
Family caregiver	Neuroticism	2.16	0.61	0.11-42.24
	Extraversion	0.40	0.35	0.06-2.70
	Openness to experience			
	Agreeableness	0.90	0.86	0.29-2.84
	Conscientiousness	0.91	0.90	0.21-4.02

 The personality trait of openness to the experience of older people/caregivers was moderate, which means that while they are not absolutely conservative, they are not very interested in new topics either. In other words, their desire for new experiences is balanced (Table 3).

 In terms of agreeableness, older people/caregivers were evaluated at the medium level; although they did not always disagree with the opinions of others, they did not agree with them much. In fact, there was a medium level of agreeableness among older people/caregivers in this study. A small number of older people/caregivers showed incompatibility; these people experienced more conflict in interpersonal relationships, the possibility of incompatibility between older people and caregivers was much greater than that between older people and caregivers, and the possibility of EA was greater for this group (Table 3).

 In the conscientiousness item, the findings showed that even though some of the older people/caregivers are responsible, law-abiding, and older people, most of them have a medium personality. Although they are not irresponsible or law-abiding people, they may have problems with this issue; therefore, sometimes they may face problems in planning and solving the problems that arise (Table 3).

 In the present study, there was a significant relationship between EA and several items related to older people’s personality traits (neuroticism, extraversion, agreeableness) at the crude analysis level (Table 3). The logistic regression model showed that high emotional stability (low neuroticism) was a protective factor against elder abuse, and older people with high emotional stability were less likely to experience elder abuse (OR = 12.05, 95% CI: 1.37-106.42) and vice versa. Additionally, a significant relationship was found between the personality traits “extroversion” and “agreeableness” and between these two traits and elder abuse (OR = 6.07, 95% CI: 1.60-23.03 and OR = 0.2, 95% CI: 0.06-0.65, respectively), which indicated that a high score for older people in these two areas was associated with a lower probability of elder abuse.

 At the crude analysis level, the logistic regression model showed that the “extroversion” of older people and caregivers had an inverse effect on EA (OR = 0.14, 95%CI: 0.02-0.90). Moreover, there were significant relationships between older people’ neuroticism and agreeableness and between older people and EA. This means that older people with high levels of extroversion and agreeableness experienced less EA, and they had high neuroticism scores.

 As mentioned above, there was an inverse relationship between caregiver extroversion and EA. In addition, there was no significant relationship between the personality traits of agreeableness (OR = 0.44, 95% CI: 0.08-2.36), conscientiousness (OR = 0.66, 95% CI: 0.23-1.89), or neuroticism (OR = 0.13, 95% CI: 0.54-137.79) and the incidence of EA. Moreover, there was no relationship between openness to experience and other personality traits in the two groups of older people or family caregivers (Table 3).

Table 4 shows the adjusted analysis table. After controlling for the variables mentioned in Table 1 (age, sex, employment status, education level, and type of residence), multivariable analysis revealed that there was no significant relationship between EA and older people/caregivers’ personality traits (Table 4).

## Discussion

 This study was conducted with the aim of determining the prevalence of self-reported domestic EA and its relationship with the personality traits of older people and their family caregivers. The mean age of the older participants was 70.10 ± 8.18 years. This study showed that the majority of older people were married (80.56%) and had their own homes (86.57%). These findings were confirmed by other studies^85^ Sotoudeh et al also confirmed that most of the older people were married and lived in their own homes.^46^

 In the present study, the prevalence of EA at home was 37.78%. Many studies have reported different prevalence rates in the last five years. The prevalence of EA reported by different researchers in Iran ranges from 34.2% to 77.9%.^45,46,55,86,87^ The lower rate of EA reported in Western research compared to Iranian research can be seen as a result of cultural factors such as social etiquette, the importance of the family institution and the role of parents in Eastern, Iranian and Islamic cultures, which leads to greater expectations of good behavior toward older people, so they consider any shortcoming as EA.

 The results showed that older people of older age and/or female gender experienced more abuse by caregivers in their families. The results of other studies also confirmed that elderly female were abused more than male elderly individuals.^4,44,46,88-91^ In contrast, a study by Khalili showed that EA is more common in the elderly men.^56^ In the present study, the older the elderly person was, the greater the amount of EA reported. Many studies have confirmed that older age is a risk factor for EA.^44,56-58^

 Additionally, unemployed older people, housewives, self-employed individuals, and retired older people were exposed to caregiver abuse in the family in descending order. Notably, unemployed older people were most likely to be exposed to abuse, and retired older people had experienced caregiver abuse less than others had. Given that unemployed older people were financially dependent on others, they experienced more EA. This result is consistent with the findings of other studies showing that the lower the older people’s income/the less skilled the individual is, the more EA occurs.^87,91^

 The illiterate older people experienced caregiver abuse in the family more than their educated counterparts. Other studies confirmed that a low education level was a risk factor for increased incidence of EA.^47,75,85,87,90-91^ This finding is not consistent with those of other studies.^44,89^ This may be because educational level is important for promoting health behaviors, seeking social support, obtaining rights, and stating opinions.

 Unmarried/single older people reported greater experiences of abuse by caregivers than married older people. Other studies confirmed this finding.^28,56^ This could be due to married older people having a support source. Older people who were tenants experienced much more abuse from their caregivers than did their counterparts who owned houses. This finding is not consistent with the study by Kissal et al which reported that there was no relationship between elder abuse and homeownership.^90^

 In the present study, psychological abuse had the highest frequency among the types of EA (25.13%), followed by other types, in descending order, of financial abuse (21.76%) and deprivation of choice (20.37%). Care neglect was 14.89%, emotional abuse was 13.82%, financial neglect was 10.25%, physical abuse was 7.05%, and rejection was 6.81%. Other studies have reported variable frequencies of EA sub-items. Other studies have confirmed that the most common abuse applied to older people in their families is psychological abuse.^28,85,86,88,90,91^ In contrast, some studies have reported care neglect as the most common EA applied.^44-45,87,92^ These differences may be due to people’s views, social etiquette, and different perceptions and attitudes toward people in different societies. Lee et al reported that the most prevalent and recognized form of EA might be psychological abuse among Asian older people.^68^ A study by Papi et al showed that the prevalence of EA among older people was 55.2%. In that study, the sample was taken from older people who were referred to the social security clinic, which could be the reason for the high prevalence.^93^

 In the present study, rejection and physical abuse were the least prevalent, with a minor difference. The findings of other studies confirm that the least common elder abuses are physical abuse and rejection.^28,44,46,56,58,85,87,94^ In the national and religious culture of Iran, physical EA is severely punishable, and many people are afraid or reluctant to engage in this type of violence.

 In the present study, there was an inverse and significant relationship between the personality traits of neuroticism and extroversion, conscientiousness, and agreeableness. Li et al showed an inverse and significant relationship between the personality trait of neuroticism and conscientiousness.^60^

 The status of the personality traits of older people and their family caregivers was investigated. At the crude analysis level, the logistic regression model showed that the “extroversion” of older people and caregivers had an inverse effect on EA. In other words, caregivers/elders with high extraversion scores had less EA (P < 0.05). People who score high in extroversion are more likely to seek social support in stressful situations and are more sociable.^95^ Extroversion may lead to optimistic expectations, and high scores of neuroticism and low extraversion may cause less coping and positive strategies. High neuroticism scores and low extraversion may cause less coping and positive strategies when encountering potentially stressful situations, which can lead to distress.^95,96^ Therefore, it can be concluded that seniors with high extroversion can manage tensions in their relationships and react appropriately in stressful situations that prevent violence in relationships, and caregivers who have high extroversion also act more rationally in the face of problems. They try to communicate with older people and solve challenges properly.

 Moreover, in the present study, older people with high agreeableness experienced less EA, and those with high neuroticism scores experienced more EA. Perry et al reported that people with neuroticism personality traits are more susceptible than other people to experiencing life-threatening and distressing events.^95^ A study by Rahimzadehsani et al confirmed that agreeableness, extroversion, and conscientiousness are significantly inversely related and that neuroticism is directly related to EA.^55^ People with high agreeableness have less tendency towards conflict in their relationships and can better control emotions such as anger, discomfort, or anxiety, which reduces tension in a person’s relationships.^97^ Steiner et alreported that people with high agreeableness and low neuroticism tend to forgive others.^98^ It improves the relationship between older people and their family and friends.

 In the present study, there was no significant relationship between conscientiousness traits in older people and EA. In contrast, the study by Li et al showed a significant and inverse relationship between conscientiousness and EA.^60^

 The present study showed that there was no significant relationship between the personality traits agreeableness (*P *˃0.05), conscientiousness (*P* ˃ 0.05), or neuroticism (*P* ˃ 0.05) and the incidence of EA. Moreover, there was no relationship between openness to experience and other personality traits in the two groups of elderly individuals and family caregivers (Table 3). In contrast, Fang et al showed that over a two-year study period, family caregivers’ neuroticism was associated with increased EA.^59^ This difference may be related to the difference between the periods in the present study and Fang’s study, in which the 2-year period of time is less likely for family caregivers to respond to the correct items of the questionnaire.

 Multivariate analysis after controlling for the variables mentioned in Table 1 (age, gender, employment status, education level, and type of residence) revealed that there was no significant relationship between EA and the personality traits of older people/caregivers. A related study with this finding was not found. However, However, After controlling for these variables, gender, age, education, marital status, income, living arrangement, depressive symptoms, years in the United States, cognitive function, medical comorbidities, and number of children, Li et al reported that there was a significant and direct relationship between neuroticism and the risk of EA. showed that there was a significant and direct relationship between neuroticism and the risk of EA.^60^ This difference may be because in Li’s study, only two personality dimensions were measured, but in the present study, we considered five dimensions of personality traits using the same questionnaire.

 This study had several limitations, such as the sampling process during the COVID-19 lockdown period, lack of data on family caregivers’ demographic characteristics, and the fact that the study was conducted in a province with a specific ethnic culture, which may limit the generalizability of the findings. Moreover, self-reported EA may be underreported in such events due to personal, cultural, and social factors. Since the current research was conducted on older people who referred to health care centers, who mostly enjoy relative health and a higher level of social participation, the actual prevalence of EA may be even greater. Therefore, caution should be taken in the generalization of the findings of this research to the society as a whole.

## Conclusion

 Due to the complexity of the issue of elder abuse and the complexity of social and cultural issues, there are a variety of statistics and numbers. Although older citizens are respected in the Iranian culture, EA is taboo and a hidden issue in Iran. In the present study, older age, female gender, unmarried/single status, lower education, unemployment, and rented house characteristics were predictors of EA. Moreover, the results showed that personality traits are related to the infrastructure of older people’ relationships with family caregivers, and it can help to predict that older people may be subjected to EA. It can be concluded that the personality traits of high agreeableness, extroversion, and low neuroticism reduce conflict and tension in the relationships of older people with their relatives and family, which appear to be protective factors against EA.

 Therefore, it seems necessary that policymakers and health experts prepare training and screening programs to take into account the abovementioned factors so that older people exposed to EA can be identified more quickly and early interventions can be used to improve their health status and increase their quality of life. In addition, guiding help-seeking and context-based standardized EA assessment tools for older people is necessary. Health policymakers should strengthen medical and social service programs for the prevention, diagnosis, evaluation, and appropriate intervention of EA in the Iranian society. When defining policies to consider EA, cultural aspects must be addressed.

## Endnote

^[1]^ R = reverse scoring with ranges from 1 (completely agree) to 5 (completely disagree).
